# PIF4 and PIF4-Interacting Proteins: At the Nexus of Plant Light, Temperature and Hormone Signal Integrations

**DOI:** 10.3390/ijms221910304

**Published:** 2021-09-24

**Authors:** Yang Xu, Ziqiang Zhu

**Affiliations:** 1 Jiangsu Key Laboratory for Biodiversity and Biotechnology, College of Life Sciences, Nanjing Normal University, Nanjing 210023, China; xuyang150720@163.com; 2 Key Laboratory of Molecular Design for Plant Cell Factory of Guangdong Higher Education Institutes, Department of Biology, Institute of Plant and Food Science, Southern University of Science and Technology, Shenzhen 518055, China

**Keywords:** PIF4, thermomorphogenesis, photomorphogenesis, protein-protein interactions

## Abstract

Basic helix-loop-helix (bHLH) family transcription factor PHYTOCHROME INTERACTING FACTOR 4 (PIF4) is necessary for plant adaption to light or high ambient temperature. PIF4 directly associates with plenty of its target genes and modulates the global transcriptome to induce or reduce gene expression levels. However, PIF4 activity is tightly controlled by its interacting proteins. Until now, twenty-five individual proteins have been reported to physically interact with PIF4. These PIF4-interacting proteins act together with PIF4 and form a unique nexus for plant adaption to light or temperature change. In this review, we will discuss the different categories of PIF4-interacting proteins, including photoreceptors, circadian clock regulators, hormone signaling components, and transcription factors. These distinct PIF4-interacting proteins either integrate light and/or temperature cues with endogenous hormone signaling, or control PIF4 abundances and transcriptional activities. Taken together, PIF4 and PIF4-interacting proteins play major roles for exogenous and endogenous signal integrations, and therefore establish a robust network for plants to cope with their surrounding environmental alterations.

## 1. Introduction

As sessile organisms, plants have to coordinate their growth and development with environmental changes, therefore enhancing their fitness and survival rates. Light and temperature are two pivotal exogenous cues for plants. Light not only provides energy for photosynthesis, but also regulates almost all of the developmental processes in the whole plant life cycle, from seed germination to flowering and senescence [1]. Seedlings grown in complete darkness undergo skotomorphogenesis, which is characterized by long hypocotyls, closed yellow cotyledons, and forming apical hooks. After light exposure, plants exhibit shortened hypocotyls and expanded green cotyledons, which is termed photomorphogenesis [2]. High ambient temperature also affects plant architecture. When temperature rises from 22 °C to 28 °C, *Arabidopsis thaliana* displays hypocotyl and petiole elongations, leaf upward growth, and early flowering, which are collectively named thermomorphogenesis [3].

To perceive and respond to exogenous environmental cues, plants elegantly modulate their endogenous phytohormone levels and/or signaling activities to promote or restrict cellular behaviors, and finally change the plant growth patterns. During the cross-talk between environmental cues and hormone signaling, basic helix-loop-helix (bHLH) transcription factors PHYTOCHROME-INTERACTING FACTORs (PIFs) play central roles. There are eight PIF members (PIF1-8) in the *A. thaliana* genome [4,5]. Among them, PIF4 plays crucial functions, which not only integrates distinct environmental and endogenous signals, but also interacts with a bunch of proteins to regulate a series of downstream responses.

In this review, we focus on the already-published PIF4-interacting proteins (Table 1), and discuss their roles in the integration of plant hormone responses with light or temperature signaling. We also showcase the recent advances on the understanding of how plants elaborately modulate PIF4 activity through PIF4-interacting kinase, E3 ubiquitin ligase, and/or transcriptional regulators. We propose that the multiple protein–protein interactions among PIF4 and PIF4-interacting proteins will generate a robust network for plants to respond to subtle light or temperature changes.

## 2. Brief History of PIF4

After the molecular cloning and characterization of red/far-red light photoreceptors phytochromes (phy), the sought-for phytochrome-interacted proteins are crucial for understanding phytochrome signaling. Early in 1998, PHYTOCHROME-INTERACTING FACTOR3 (PIF3) was successfully identified through a yeast two-hybrid screening [24]. PIF3 belongs to the basic helix-loop-helix (bHLH) transcription factor family and has two conserved motifs (Active phytochrome A-binding motif (APA) and Active Phytochrome B-Binding (APB) motif), which mediate its interaction with phyA or phyB, respectively [25,26]. Later, in a genetic screen for identifying new components in the phytochrome signaling pathway, one T-DNA insertion mutant *srl2* (*short under red light 2*) was found to be highly sensitive to red light and its corresponding gene *SRL2* encoded a PIF3-like bHLH transcription factor, which was therefore named PIF4 [6]. PIF4 also directly interacts with the bioactive Pfr form of phyB through its APB motif. Although PIF4 does not have an APA motif that is necessary for interaction with phyA, it still interacts with phyA with a lower affinity than phyB [6].

As a key transcription factor in light and temperature, PIF4 binds to thousands of target genes to regulate their expressions. For example, when ambient temperature elevates, PIF4 proteins accumulate and associate with its target gene promoters (such as *YUCCA8* (*YUC8*) and *INDOLE-3-ACETIC ACID INDUCIBLE 19* (*IAA19*)) to upregulate auxin biosynthesis and signaling, which cause cell elongation and plant growth [27,28,29,30].

## 3. PIF4-Interacting Photoreceptors

### 3.1. Phytochromes

**Figure 1:**
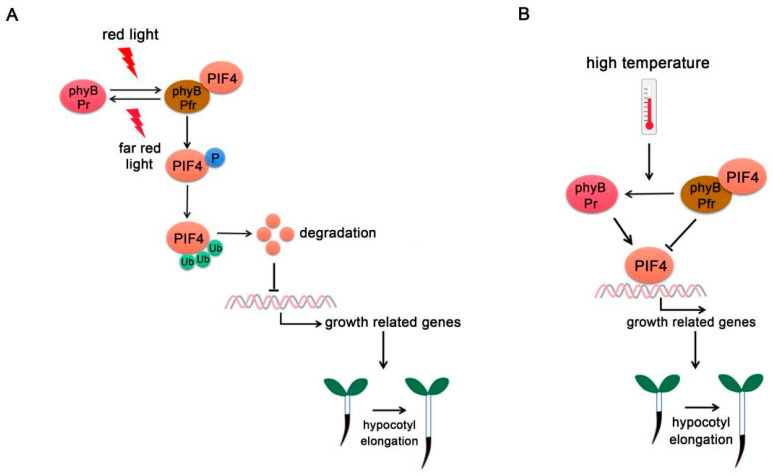
**PIF4-interacting phytochromes.** (**A**) **PhyB regulates PIF4 activity under red/far-red light.** At far-red light, phyB exists in the inactive Pr form. Upon red light irradiation, phyB transforms into the biologically active Pfr form and interacts with PIF4, thus initiating PIF4 phosphorylation (P), ubiquitination (Ub), and degradation. (**B**) **Phy B regulates PIF4 activity at high temperature.** High ambient temperature promotes the phyB reversion from Pfr form into Pr form and releases its repression on PIF4. PIF4 accumulates and binds to the promoters of growth-related genes to promote their expressions. In addition, phyB directly binds to the promoters of growth-related genes and promotes hypocotyl elongation.

PIF4 was first identified as a negative regulator of the phytochrome signaling pathway. Phytochromes perceive red and far-red light and promote photomorphogenesis through complex regulatory mechanisms [31,32]. In the dark, phyB exists in the biologically inactive Pr form, while PIFs accumulate in the nucleus and regulate gene expressions that inhibit photomorphogenesis. While under red light, phyB transforms into biologically active Pfr state and interacts with PIF4. Direct physical interaction between PIF4 and phytochromes causes light-induced phosphorylation followed by ubiquitylation and subsequent degradation of PIF through the 26S proteasome-mediated protein degradation (Figure 1A) [33]. PIFs degradation is regulated by E3 ubiquitin ligase. Different E3 ligases regulate the degradation of distinct PIFs. For example, Bric-a-Brack/Tramtrack/Broad (BTB)-Cullin3-type E3 ubiquitin ligase LIGHT RESPONSE BTB (LRB) proteins directly interact with the red light-activated phyB-PIF3 complex and subsequently ubiquitinate both phyB and PIF3 for degradation [34]. The degradation of both phyB (light receptor) and PIF3 (its immediate signaling partner) weakens light signaling, thereby preventing unnecessary light responses. In addition, the phyB-PIF interaction reciprocally triggers degradation of phyB under red light, forming a negative feedback loop [34,35].

In addition to being a photoreceptor, phyB also functions as a thermosensor (Figure 1B). High temperature promotes Pfr-phyB converted into the Pr form and facilitates PIF4-induced cell elongation [3]. *phyB-1* mutants show constitutively long hypocotyl phenotypes at a temperature range from 12 °C to 27 °C. Temperature changes the phyB nuclear body formation and the (indirect) association of phyB to promoters of key target genes related to plant growth such as *YUC8* or *AUXIN RESPONSE FACTOR7* (*ARF7*). These effects are caused in part by the modified activity, but the rates of association and dissociation could also be directly influenced by temperature [36,37,38].

### 3.2. Cryptochromes

**Figure 2:**
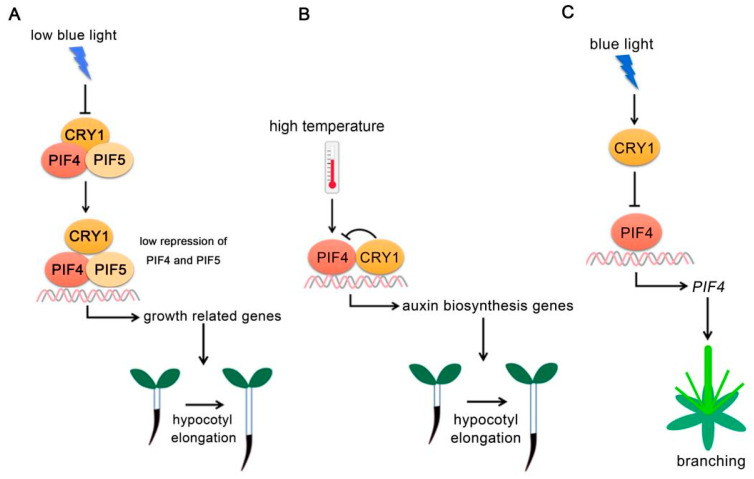
**PIF4-interacting cryptochromes.** (**A**) **CRY1 regulates PIF4 and PIF5 activity under low blue light.** Under low blue light, CRY1 activity decreased and its interaction with PIFs was reduced, allowing PIFs to activate growth-related gene expressions. (**B**) **CRY1 regulates PIF4 activity at high temperature.** At high ambient temperature, CRY1 directly interacts with PIF4 and abrogates PIF4 DNA-binding activity to repress auxin biosynthesis-related gene expressions. (**C**) **CRY1-PIF4 module regulates plant branching.** CRY1 abrogates PIF4 binding to its own promoter. PIF4 positively regulates plant branching.

Cryptochrome 1 (CRY1) and CRY2 are blue light photoreceptors in *Arabidopsis* [7,39,40]. CRY1 mainly regulates the inhibition of hypocotyl elongation under blue light, while CRY2 mainly functions in photoperiod flowering [41]. In order to avoid being covered by neighboring plants, plants have evolved a series of adaptive characteristics, which are called shade-avoidance response (SAR). Studies in different species have revealed that reduced or low blue light (LBL) can cause shade-avoidance response [42,43,44]. CRY1 regulates the LBL-triggered SAR, partially depending on the physical interaction with PIF4 and PIF5 [45,46]. Under low blue light, the activity of CRYs decreased and their interaction with PIFs weakened, allowing PIFs to bind to the promoters of genes promoting plant growth in order to facilitate hypocotyl elongation (Figure 2A) [46]. In addition to SAR, CRY-PIFs interactions also regulate high temperature-mediated hypocotyl elongation. CRY1 directly interacts with PIF4 in a blue light-dependent manner and represses the expressions of auxin biosynthesis-related genes under high ambient temperature, thereby inhibiting thermomorphogenesis under blue light conditions (Figure 2B) [29].

A recent study also demonstrate that the CRY1-PIF4 module participates in the regulation of plant branching architectures. *cry1* mutants show increased branching phenotypes. Furthermore, *PIF4* expression levels are elevated in the *cry1* mutant. PIF4 binds to the G-box motif of the *PIF4* promoter and forms a self-activated positive feedback loop, while CRY1 represses this process under blue light [47].

## 4. PIF4-Interacting Circadian Clock Components

### 4.1. ELF3

**Figure 3:**
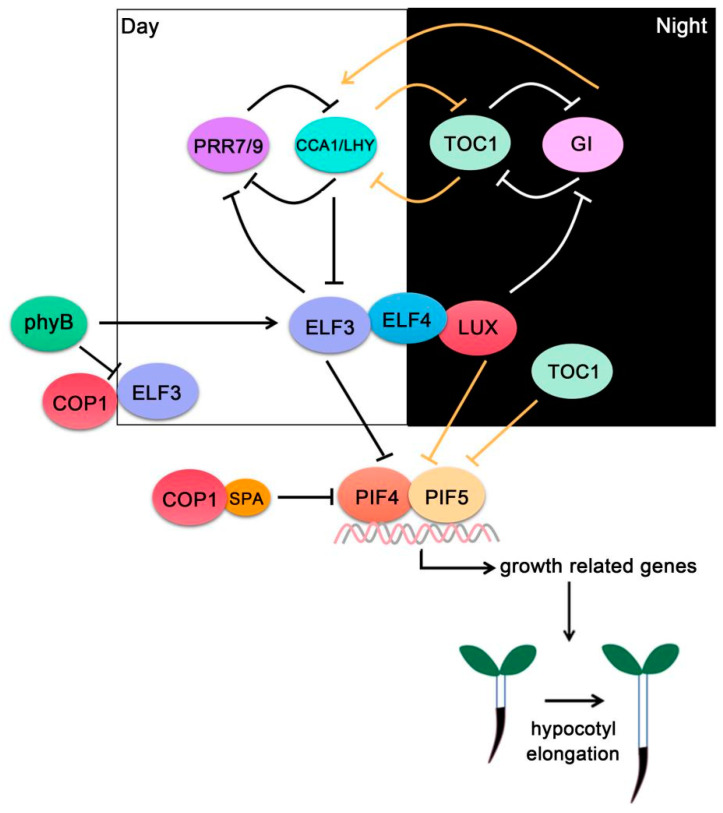
**PIF4-interacting circadian clock components** ELF3, ELF4 and LUX compose the evening complex (EC), which interacts with PIF4/PIF5 and represses their transcriptional activities. The circadian clock controls EC activity through a series of transcriptional and post-translational feedback loops.

The circadian clock governs plant daily behaviors and is also crucial for maintaining plant fitness. Plant growth is regulated by the intrinsic circadian clock and light entrainment [48,49,50,51]. In the photoperiodic growth, CIRCADIAN CLOCK-ASSOCIATED 1 (CCA1) and LATE ELONGATED HYPOCOTYL (LHY) peak at dawn and repress TIMING OF CAB EXPRESSION1 (TOC1) and evening complex (EC) expressions in the morning. TOC1 also represses the transcription of the EC complex [52]. Meanwhile, CCA1/LHY activate PSEUDORESPONSE REGULATOR (PRR) expressions. From dawn to dusk, PRR9/7 suppress the expression of *CCA1/LHY* [53]. In the evening, EC negatively regulates PRR9 to release their inhibition of *CCA1*/*LHY* and promotes *CCA1*/*LHY* expression peaks in the early morning [52,54]. In Arabidopsis, ELF3, ELF4, and LUX proteins compose the EC complex, which is indispensable for the normal expression of *PIF4* and *PIF5* under diurnal conditions (Figure 3). EC complex directly binds to *PIF4* and *PIF5* promoters in vivo. Mutations in *PIF4* and *PIF5* are epistatic to the loss of the ELF4-ELF3-LUX complex, suggesting that one of the most significant functions of this complex is to regulate *PIF4* and *PIF5* expressions. Further research shows that the circadian-regulated EC represses *PIF4* and *PIF5* expression in the evening. During the day, light-mediated PIF4 and PIF5 protein degradation inhibits growth, while near dawn, the concomitant rise in *PIF4* and *PIF5* mRNA and PIF4 and PIF5 protein levels promotes growth (Figure 3) [55].

In addition, further research showed ELF3 interaction with PIF4 independently of EC function [16]. PIF4 overexpression causes ELF3 protein destabilization, and this process is mediated indirectly by negative feedback regulation of photoactive phyB [16]. In the light, photoactivated phyB is translocated into the nucleus and promotes ELF3 accumulation, probably through the disruption of CONSTITUTIVELY PHOTOMORPHOGENIC1 (COP1)-ELF3 interactions. ELF3 binds the PIF4 bHLH domain in an EC-independent manner and prevents PIF4 from binding to its DNA recognition sequences (Figure 3) [16].

### 4.2. PRRs

PRRs are necessary transcription factors in the plant circadian clock. There are five PRRs in Arabidopsis, including PRR3, PRR5, PRR7, PRR9, and TOC1 (also named PRR1). PRRs coordinate with EC to specifically regulate photoperiodic hypocotyl growth [56,57]. Studies have shown that all the five PRRs interact with PIF4 [17]. The expressions of PIF4 target genes display diurnal rhythms of thermosensitivity. When transferred from 20 °C to 29 °C for 4 h during ZT 0-ZT 4 (zeitgeber time, ZT), the *PIF4* mRNA levels only show a 25% increase, but *YUC8* RNA levels display over a three-fold increase. In contrast, high-temperature treatment during ZT 8-ZT 12 and ZT 12-ZT 16 increases *PIF4* RNA levels over five-fold, but does not change *YUC8* expression levels obviously [17]. TOC1 inhibits PIF4 activity and suppresses thermoresponsive growth in the evening by preventing PIF4 from binding to its targets. Loss of function of *TOC1* and its close homologue *PRR5* restores thermosensitivity in the evening, whereas TOC1 overexpression leads to thermoinsensitivity, indicating that TOC1 specifically inhibits thermoresponses in the evening (Figure 3) [17].

In addition, PRRs regulate photoperiodic hypocotyl growth by directly regulating *PIF4* and *PIF5* transcriptions [58]. A distinct daylength can alter the expression pattern and extend the expression duration of *PRRs*. PRRs function as transcriptional repressors of *PIF4* and *PIF5*, which directly bind to the promoters of *PIF4* and *PIF5* to inhibit their expressions [58]. Moreover, mutation or truncation of the TOC1 DNA binding motif, without damaging its interaction with PIFs, still causes long hypocotyl growth under short days, demonstrating the essential roles of the PRR-PIF transcriptional module in photoperiodic hypocotyl growth [58].

## 5. PIF4-Interacting Plant Hormone Signaling Components

### 5.1. Abscisic Acid (ABA)

ABA mainly regulates plant development and particularly response to abiotic stresses [59,60]. The core ABA signaling components include ABA receptors (PYRABACTIN RESISTANCE 1 (PYR1) and PYR1-Like (PYL) proteins, also named REGULATORY COMPONENTS OF THE ABA RECEPTORS (RCARs)), protein phosphatase 2Cs (PP2Cs), and SNF1-related protein kinase 2s (SnRK2s) [61,62,63]. In the absence of ABA, PP2Cs repress SnRK2 activity and downstream ABA responses, while in the presence of ABA, ABA binds to PYR/PYL/RCARs receptors and forms a coreceptor complex with PP2Cs and represses their phosphatase activity. Then, the SnRK2s kinase activity is released and phosphorylate their target proteins [61,62,63].

**Figure 4:**
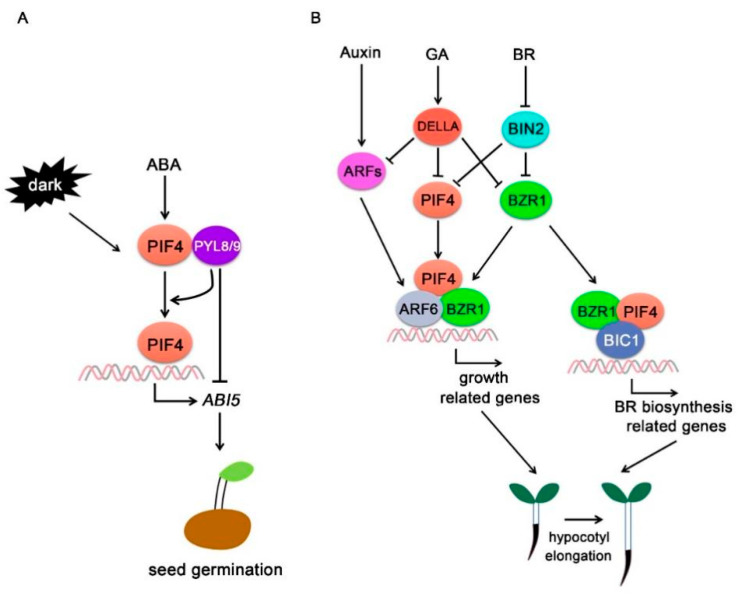
**PIF4-interacting hormone signaling components** (**A**) **PIFs specifically regulate ABA signaling in the dark.** ABA promotes *PIF4* activity in the dark, and PIF4 interacts with PYL8 and PYL9 in an ABA-independent manner. PYL8 and PYL9 promote PIF4 binding to the *ABI5* promoter but inhibit PIF4-mediated *ABI5* activation. (**B**) **PIF4 integrates auxin, GA, and BR signals to regulate plant growth.** ARF6 interacts with PIF4 and BZR1 and forms a BAP module to regulate lots of their common targets and promote hypocotyl elongation. GA signaling repressor DELLA proteins interact with PIF4 and inhibit PIF4 activity and stability. BZR1 and BIC1 function as transcriptional coactivator and interact with PIF4 to regulate BR biosynthesis gene expressions and hypocotyl elongation.

Recently, it was reported that PIF4 positively regulates ABA signaling specifically in the dark. When treated with ABA, *pifq* (*pif1345* quadruple) mutants show higher germination rates than Col-0, and the ABA-induced primary root growth inhibitions in *pifq* are weaker than in Col-0. PIF4 directly associates with the *ABI5* promoter and positively regulates ABA-mediated *ABI5* transcription and protein accumulations. ABA promotes *PIF4* gene expression in the dark, and PIF4 interacts with PYL8 and PYL9 in an ABA-independent manner. PYL8 and PYL9 facilitate PIF4 to bind to the *ABI5* promoter, but inhibit PIF4-regulated activation of *ABI5* transcription (Figure 4A) [8].

### 5.2. Gibberellins (GAs)

GAs plays significant roles in plant growth and development, which control seed germination, hypocotyl elongation, and flowering time [64]. DELLA proteins are repressors in GA signaling. There are five DELLA proteins in Arabidopsis: GA-INSENSITIVE (GAI), REPRESSOR OF GA1-3 (RGA), RGA LIKE1 (RGL1), RGL2, and RGL3 [64,65]. In the absence of GA, DELLA proteins interact with various transcription factors or transcriptional regulators and inhibit their activities, while in the presence of GA, the GA receptor GIBBERELLIN INSENSITIVE DWARF1 (GID1) binds to DELLAs and forms the GID1-GA-DELLA complex, which further triggers the ubiquitination and subsequent degradation of DELLA proteins through the 26S proteasome to relieve their inhibition of the transcription factors and cause a serious of GA responses [64].

GA antagonizes light signaling during plant growth [66,67,68]. GA induces the degradation of DELLA proteins [69], but light promotes the accumulation of DELLA proteins through reducing GA contents [70]. DELLA interacts with both PIF3 and PIF4 and regulates their activities. In the absence of GA, nuclear-localized DELLA proteins accumulate and interact with PIF3 to prevent PIF3 binding to its target genes, which therefore abrogates the PIF3-mediated light control of hypocotyl elongation. In the presence of GA, GID1 proteins enhance their interaction with DELLA proteins in the nucleus and triggers DELLA protein degradation. With the degradation of DELLA repressors, PIF3 activities are derepressed [71]. Similarly, PIF4 is destabilized by phyB in the light and DELLAs inhibit PIF4 transcriptional activity via binding to its DNA-recognition domain. GAs release such inhibition through promoting DELLA protein turnover, and therefore cause a subsequent activation of PIF4 in the nucleus [12].

In addition to the regulation of PIF transcriptional activity, DELLA proteins also control PIF protein stability. PIF3 protein levels increases obviously in the *della* pentuple mutants, and induction of DELLAs promotes PIF3 degradation [72]. Taken together, DELLA proteins inhibit PIF activities through two pathways: (1) promoting PIF degradation through the 26S proteasome and (2) sequestrating PIFs from associating with its target genes. Application of GA will cause DELLA degradation and further release their inhibitions on PIFs, which nicely coordinate plant growth under certain environments (Figure 4B).

### 5.3. Brassinosteroid (BR)

BR mainly regulates plant hypocotyl elongation, photomorphogenesis, and flowering time [73,74,75,76,77]. BR-deficient mutants display dwarfism and dark-green color cotyledon phenotypes when grown in light, and exhibit de-etiolation phenotypes when grown in darkness [78,79,80,81]. In the past decades, the major components and signaling mechanisms of BR have been revealed. BR binds to its receptor kinase complex BRASSINOSTEROID-INSENSITIVE 1 (BRI1) and BRI1-ASSOCIATED PROTEIN KINASE 1 (BAK1), then the complex phosphorylates its downstream components. BRI1 phosphorylates BRASSINOSTEROID SIGNALING KINASE 1 (BSK1) and CONSTITUTIVE DIFFERENTIAL GROWTH 1 (CDG1), which phosphorylate BRI1-SUPPRESSOR 1 (BSU1). BSU1 dephosphorylates the glycogen synthase kinase 3 (GSK3)-like kinase BRASSINOSTEROID INSENSITIVE 2 (BIN2) [82,83]. When BR is absent, BIN2 is active and phosphorylates transcription factors BZR1 and BRI1-EMS-SUPPRESSOR 1 (BES1). While in the presence of BR, dephosphorylated BIN2 is inactive. Therefore, BZR1 and BES1 are dephosphorylated and subsequently moved into the nucleus to control BR-responsive gene expressions [84,85].

BR integrates different environmental cues such as light or temperature to control cell elongation. BZR1 directly interacts with PIF4 and controls a subset of overlapped downstream gene expressions [10]. In addition, the BZR1-PIF4 module promotes plant growth in response to BR, darkness, or high temperature [9]. High temperature increases PIF4 protein accumulations and promotes the formation of PIF4-BES1 complex and then activates the expressions of BR biosynthesis-related genes [86]. The increase in BR level induces hypocotyl growth through inactivating BIN2 and activation of PIF4 and BES1 transcription factors [86]. When exposed to light, phyB decreases PIF4 stability, and BES1 played a major role to inhibit the expression of BR synthesis-related genes and reduce BR contents. Thus, BZR1-PIF4 interaction controls a core transcription network, enabling plant growth co-regulation by the steroid hormone and environmental signals [86]. Recently, it was shown that BLUE-LIGHT INHIBITOR OF CRYPTOCHROMES 1 (BIC1) is a new BZR1-interacting protein, which functions as a transcriptional coactivator for BZR1-mediated regulation of BR signaling. Meanwhile, BIC1 interacts with PIF4 to interdependently activate the expression of downstream genes including *PIF4* itself, and to promote hypocotyl elongation by binding to the promoters of their common targets (Figure 4B) [10].

### 5.4. Auxin

Auxin plays key roles in nearly all the physiological processes, including cell elongation, shade avoidance, warm temperature response, and tropic growth responses to light or gravity [87,88]. Similar to the GA signaling, auxin signaling also belongs to the derepression mechanisms. Briefly, without auxin, Auxin response factor (ARF) transcription factors are repressed by Aux/IAA proteins through their physical interactions. After auxin treatment, auxin acts as a “molecular glue” to promote the interactions between Aux/IAA proteins with the F-box protein TIR1, which serves as auxin receptor protein. Then, Aux/IAA proteins are degraded through the 26S proteasome-mediated protein degradation pathway. The removal of Aux/IAA repressions result in the activation of ARFs and elicits auxin responsive gene expressions and auxin responses [89,90].

ARF6 physically interacts with both PIF4 and BZR1 (aka BZR1-ARF6-PIF4 (BAP) module) to synergistically regulate hypocotyl elongation and plant growth (Figure 4B). It is illustrated that 42% of ARF6-targeted genes are also targeted by both BZR1 and PIF4. The common targets of the BAP module include *EXPANSIN, SMALL AUXIN UPREGULATED* (*SAURs*), and *AUX/IAA* genes, which are mainly related to cell wall modifications and auxin responses to control cell growth [11]. The coding product of HLH transcription factor *PACLOBUTRAZOLE RESISTANT 1* (*PRE1*), which interacts and inhibits the growth repressor ILI1 BINDING bHLH PROTEIN1 (IBH1), is another common target for the BAP module. Moreover, ARF6, BZR1, and PIF4 interdependently activate shared target genes to synergistically modulate cell elongation and hypocotyl growth [11].

In addition, it is reported that *pif4-101* mutants display short hypocotyls, and auxin content in *pif4-101* decreased even under 28 °C compared with WT, while synthetic auxin picloram can rescue their short hypocotyl phenotypes, indicating that PIF4 is able to regulate auxin biosynthesis [26]. The conversion of tryptophan to indole-3-pyruvic acid is a key step in auxin biosynthesis. It was catalyzed by TRYPTOPHAN AMINOTRANSFERASE OF ARABIDOPSIS (TAA1). Then, key rate-limiting flavin monooxygenase enzymes YUCCAs (YUCCA1-11) catalyze the conversion of IPyA into IAA [91] Moreover, CYP79B2, and CYP79B3 function in a separate auxin biosynthesis pathway and convert tryptophan to indole-3-acetaldoxime. Mutants deficient in these auxin biosynthesis-related genes display impaired hypocotyl elongation response to high temperature. Expression levels of these genes are significantly induced under high temperature, and these inductions are greatly dampened in *pif4-101* mutants, suggesting a role for PIF4 in the temperature-mediated upregulation of these auxin biosynthesis-related genes. In addition, PIF4 directly binds to the promoters of all these auxin biosynthesis-related genes and promotes their expressions [26].

## 6. PIF4-Interacting Transcriptional Regulators

### 6.1. Positive Regulators

#### 6.1.1. HLS1

*HOOKLESS 1* (*HLS1*) was firstly identified in genetic screening for ethylene-insensitive mutants. Etiolated *hls1* mutants do not exhibit exaggerated apical hooks even when treated with ethylene [92]. HLS1 protein sequence is similar to the *N*-acetyltransferase in yeast or animals [93]. However, in vitro enzyme activity assay did not support HLS1 acetyltransferase activity [94]. Interestingly, *hls1* mutants exhibit hyposensitivity to high-temperature-triggered cell elongation and transcriptomic changes, which suggested that HLS1 functions as a positive regulator in thermomorphogenesis [95].

**Figure 5:**
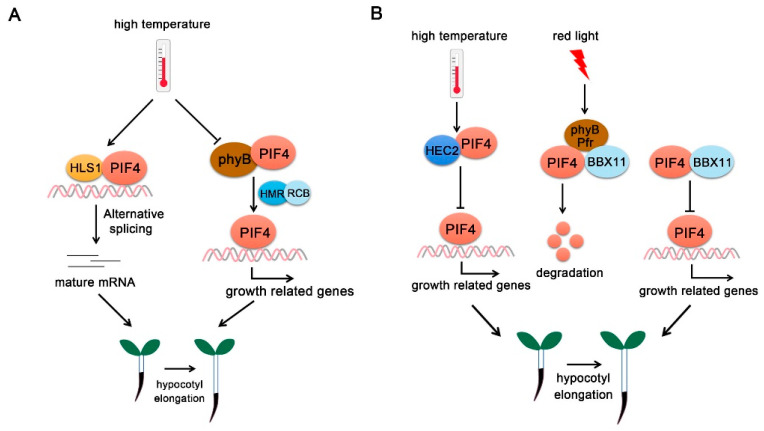
**PIF4-interacting transcriptional regulators.** (**A**) **PIF4-interacting positive regulators.** At high temperature, HLS1 interacts with PIF4 and form a regulatory module with PIF4 to collectively regulate hypocotyl elongation partially through alternative splicing. HMR and RCB collaboratively promote hypocotyl elongation via stabilizing PIF4. (**B**) **PIF4-interacting negative regulators.** At high temperature, HEC2 interacts with PIF4 and inhibits PIF4 DNA-binding activity and represses hypocotyl elongation. Under red light, BBX11 enhances PIF4-phyB interaction and promotes PIF4 degradation. In addition, BBX11 interacts with PIF4 to abrogate its association with growth-related gene promoters and inhibit hypocotyl elongation.

Moreover, HLS1 interacts with PIF4 to collectively regulate thermomorphogenesis partially through their co-regulations on differentially alternative splicing events and differentially expressed genes [19]. PIF4 and HLS1 co-regulate a large number of common target genes. Moreover, 27.7% of them are direct targets of PIF4 [19]. Thus, HLS1-PIF4 module controls both transcriptional and posttranscriptional regulations during plant thermomorphogenesis (Figure 5A) [19].

#### 6.1.2. HMR

As a thermosensor, phyB mainly functions at night, but some reports also showed that phyB plays critical roles in the daytime. In daytime thermosensing, phyB signals are mainly regulated through PIF4, which requires the transcriptional activator HEMERA (HMR) [15]. HMR is a nuclear and plastidic dual-targeted protein that involved in phyB-mediated photomorphogenesis and thermomorphogenesis [96,97,98]. Plastidic HMR is an essential component of the plastid-encoded RNA polymerase responsible for the expression of plastid-encoded photosynthesis genes [99], while nuclear HMR is a transcriptional activator that directly interacts with phyB and all PIFs [98]. Notably, HMR functions conversely on the activity of PIF3 and PIF4. HMR promotes PIF3 degradation in photomorphogenesis [96,98,100], while enhances PIF4 stabilization in thermomorphogenesis.

In the thermoresponse detection, only *pif135* triple mutants show wild-type-like thermoresponse, while the other triple *pif* mutant combinations exhibit phenotypes similar to *pifq*. Furthermore, the PIF4-dependent thermoresponse (27 °C relative to 21 °C) was reduced dramatically to 17% in *pif135/hmr-5* and 46% in *pif135/hmr-22*, indicating that the PIF4-dependent high-temperature response requires HMR. HMR does not regulate *PIF4* transcription, but interacts directly with PIF4 and activates the plant growth-related gene expressions and promotes PIF4 accumulation [15]. REGULATOR OF CHLOROPLAST BIOGENESIS (RCB), which interacts with HMR, acts as a novel temperature signaling component that functions collaboratively with HMR to initiate thermomorphogenesis by selectively stabilizing PIF4 in the daytime [101]. *rcb-101* mutants completely rescue the short-hypocotyl phenotypes of *hmr-22* mutants at 27 °C and restore PIF4 stability and activity in *hmr-22*. In addition, RCB regulates PIF4 stability and activity and is required for thermoresponsive PIF4 accumulation. Therefore, HMR and RCB collaboratively enable thermomorphogenesis via stabilizing PIF4 (Figure 5A) [101].

### 6.2. Negative Regulators

#### 6.2.1. BBX11

B-box proteins (BBXs), which are characterized by containing conserved B-box domains at their N-terminus, play significant roles in plant photoperiodic flowering, hormone responses and photomorphogenesis [102,103,104,105]. There are 32 BBXs in Arabidopsis, which are divided into five subfamilies according to their domain structures and features [106]. Increasing studies have revealed that individual BBX protein has different functions. BBX4 and BBX21-BBX23 are positive regulators for light signaling, whereas BBX19, BBX24, BBX25 and BBX28-BBX32 inhibit photomorphogenesis [20,107,108,109,110,111,112,113,114,115,116,117,118,119,120,121,122,123,124]. Some BBXs form a transcriptional regulatory network with ELOGATED HYPOCOTYL 5 (HY5) through affecting HY5 activity to regulate photomorphogenic development. For example, BBX11, a positive regulator of red light signaling, binds to *HY5* promoter to activate its transcription, while both BBX21 and HY5 associate with the promoter region of *BBX11* and positively regulate *BBX11* expression. Thus, BBX11–BBX21–HY5 form a positive feedback loop and provide an important mechanism for seeding development in response to light [125].

BBX11 also interacts with both phyB and PIF4 [14]. BBX11 enhances the interaction between phyB and PIF4. PIF4 protein accumulation and activities are significantly repressed by BBX11. When transferred from dark to red light, PIF4 protein decreased rapidly in Col-0, *bbx11* and *YFP-BBX11* plants, especially in *YFP-BBX11.* In contrast to red light, PIF4 protein accumulates more in *bbx11* mutants, indicating an inhibition role of BBX11 for PIF4 proteins [14]. Furthermore, BBX11 functions upstream of PIF4 and inhibits PIF4-regulated gene expression and finally promotes photomorphogenesis under red light (Figure 5B) [14].

#### 6.2.2. HEC2

HECs are bHLH transcription factors, which play essential roles in fertilization and photomorphogenesis [30,126]. HECs are positive regulators of photomorphogenesis, which directly interacts with PIFs and inhibits PIFs activity to form a negative feedback with PIFs. HECs are stabilized in the light and degraded in the dark through 26S proteasome-mediated protein degradation pathway. It was found that E3 ligase COP1 directly regulates HECs protein levels through ubiquitylation and degradation in darkness [127].

Recently, another elegant study showed that HEC1 and HEC2 inhibit thermomorphogenesis by forming a negative feedback loop with PIF4 [18]. *hec1 hec2* double mutants exhibited much longer hypocotyls at 28 °C compared with Col-0 controls, while *HEC2ox* plants showed short hypocotyls. Furthermore, high temperature upregulated *HEC2* expression levels and stabilized HEC2 protein levels [18]. Genetic analysis showed that *PIFs* are epistatic to *HEC2*. HECs and PIFs antagonistically control the expression of lots of genes in response to high ambient temperature. PIFs activate the expression of *HECs* at high temperature. HEC2 in turn interacts with PIF4 and forms a negative feedback loop (Figure 5B) [18].

## 7. PIF4-Interacting Kinase

SUPPRESSOR OF PHYA-105 (SPA) family was first discovered as inhibitor of phyA signaling [128,129,130]. Four *SPA* genes (*SPA1, SPA2, SPA3*, and *SPA4*) have been characterized in Arabidopsis, which play redundant roles in plant development [128,129,130]. SPAs contain a Ser/Thr kinase domain at their N-terminus, a coiled-coil domain in the middle, and four WD-40 repeats in their C-terminus serving as protein–protein interaction domains [129]. SPAs have been demonstrated to positively control COP1 E3 ubiquitin ligase activity in plant photomorphogenesis [128,131]. Recently, SPA1 was reported to have Ser/Thr kinase activity and directly phosphorylated PIF1 [132].

**Figure 6:**
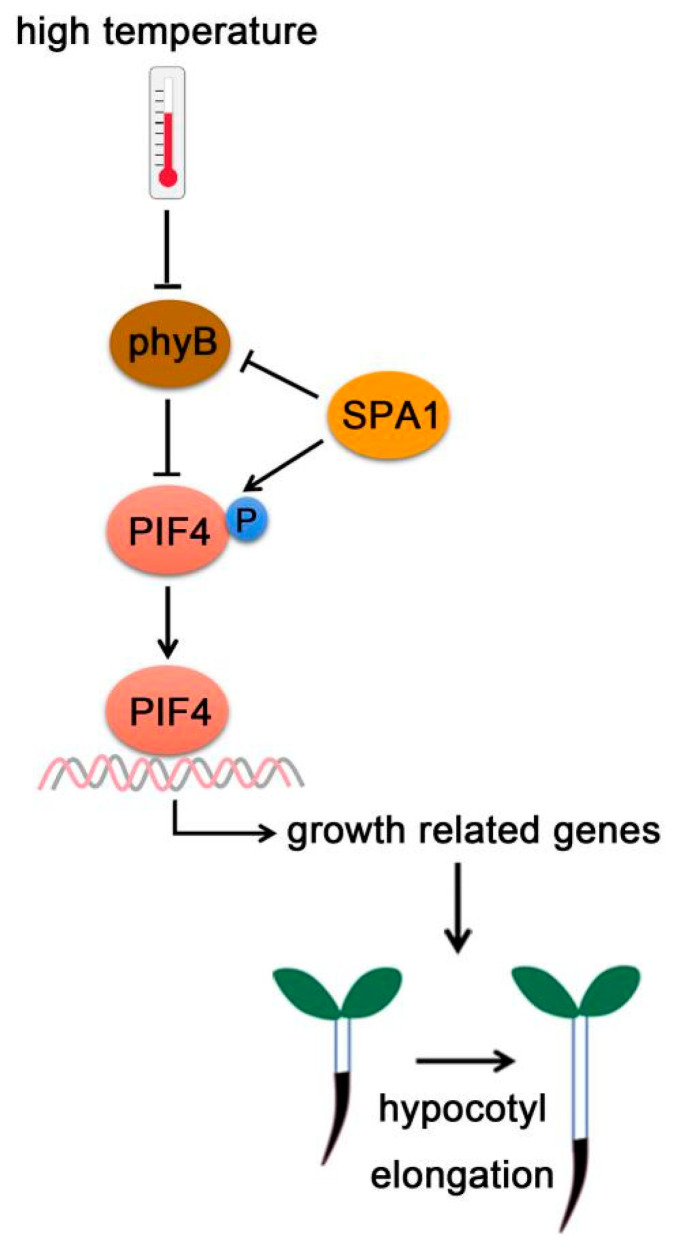
**PIF4-interacting kinase**. At high temperature, SPA1 promotes phyB degradation, but enhances PIF4 stabilization through triggering PIF4 phosphorylation. Activated PIF4 then binds to the promoters of growth-related genes and promotes hypocotyl elongation.

Meanwhile, SPAs also phosphorylates PIF4 and promotes thermomorphogenesis [13]. Under high temperature, phyB protein levels increased in *spaQ* (*spa1 spa2 spa3 spa4*) quadruple mutants, indicating that SPAs promotes phyB degradation. However, PIF4 protein levels decreased in the *35S: PIF4-Myc/spaQ*, indicating that SPAs stabilized PIF4 [13]. Further study showed that SPA1 phosphorylated PIF4 for its stabilization. Stabilized PIF4 then promotes hypocotyl elongation by binding to the promoters of growth-related genes (Figure 6) [13].

## 8. PIF4-Interacting E3 Ubiquitin Ligase

Under prolonged red light, phyB transforms into biologically active Pfr form and interacts with PIF4 to initiate PIF4 phosphorylation, ubiquitination and degradation. There are distinct E3 ubiquitin ligase regulating the degradation of different PIFs [33]. BLADE-ON-PETIOLE 1 (BOP1) and BOP2 are two homologs that contain Bric-a-Brack/Tramtrack/Broad (BTB) domain, which further complex with the cullin3 (CUL3)-based E3 ubiquitin ligase complexes (CUL3^BOP1/BOP2^). BOP1 and BOP2 were previously shown to redundantly regulate leaf development. In *bop1 bop2* double mutants, the leaf lamina extends along the petioles and leaves become massively elongated [133,134,135], which are partially reminiscent of responses to changes in light quality [136].

**Figure 7:**
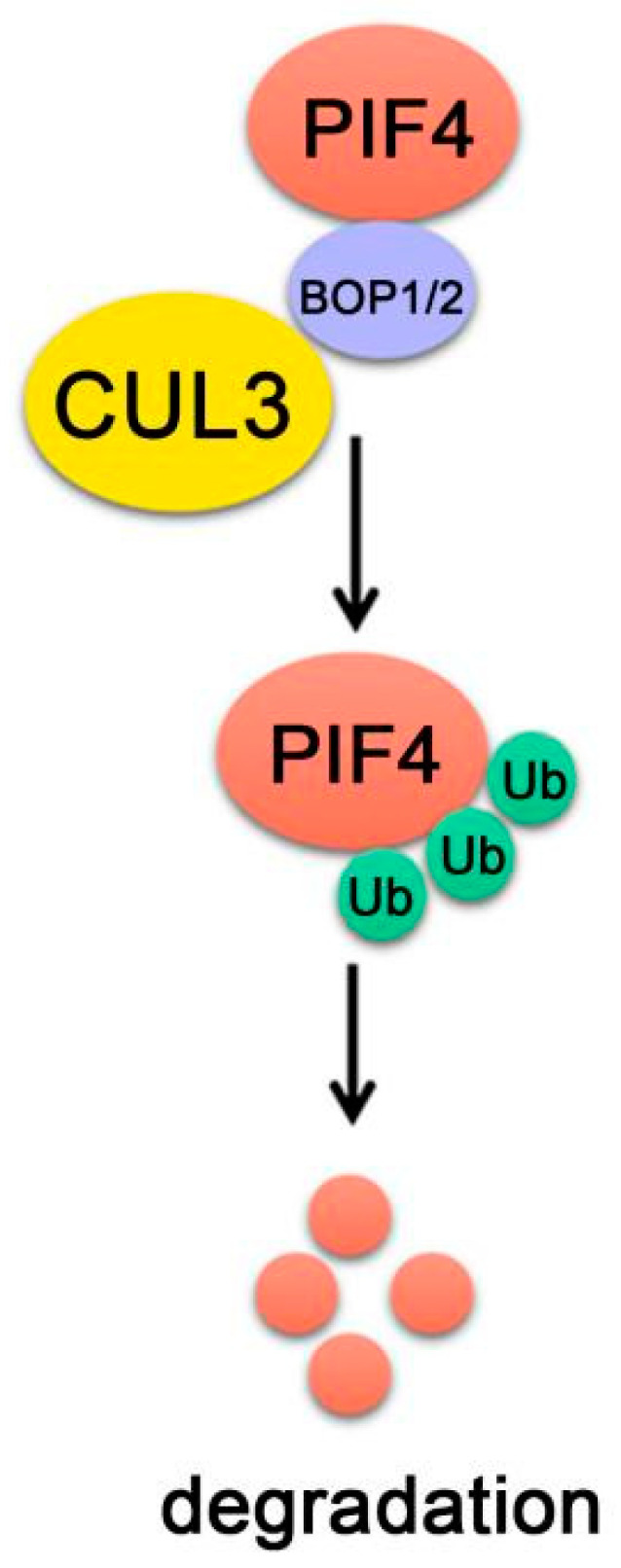
**PIF4-interacting E3 ubiquitin ligase**. BOP1/2 interact with both PIF4 and CUL3. CUL3^BOP1/2^ complex ubiquitinates PIF4 and targets PIF4 proteins turnover.

BOP1 and BOP2 are involved in the controlling of PIF4 protein abundance [20]. Genetic analysis shows that BOP2 promotes photomorphogenesis and regulates thermomorphogenesis by inhibiting PIF4 activity through reducing PIF4 protein levels. In red-light-grown seedlings, PIF4 ubiquitination was reduced in the *bop2* mutants, while PIF4 protein levels increased at both 22 ℃ and 28 ℃ [20]. Moreover, it was found that BOP proteins directly interact with both PIF4 and CUL3 and the CUL3^BOP2^ complex ubiquitinates PIF4 in vitro, indicating that BOP1 and BOP2 proteins target PIF4 for degradation (Figure 7) [20].

## 9. Perspectives

As a central regulator of light and temperature signaling, PIF4 interacts with various proteins including photoreceptors, hormone signaling components, clock components, transcriptional regulators, kinases, and E3 ubiquitin ligases. These different interactions form a robust PIF4-centered regulatory network, which can be modulated by either environmental stimuli or endogenous hormones. Although plenty of progresses have been made in the past decade, there are still several questions to be solved in the future.

Although *PIF4* is expressed in all tissues, the tissue-specific PIF4-interacting proteins are still not clear. An extremely recent study showed that over-expression of *PIF4*, specifically at the epidermis, results in long hypocotyl phenotypes [137], suggesting that the promotion effect on hypocotyl elongation is epidermis-dependent. It is intriguing to dissect the tissue-specific PIF4-interacting proteins through cutting-edge single-cell technology coupled with mass spectrometry analysis.

With the discoveries of numerous PIF4-interacting proteins in Arabidopsis, it is not too late to ask the origin of these PIF4-interacting proteins. It is plausible to test these protein–protein interactions in their homologs in algae, moss, ferns, gymnosperms, and ancient angiosperms.

As we mentioned in the previous text, these PIF4 and PIF4-interacting proteins are described one by one. We do not know when PIF4 interacts with protein A under a specific condition, whether it still interacts with protein B or not *in planta*. Therefore, it is urgent to develop a new tool for simultaneously monitoring multiple protein–protein interactions. Hopefully future studies in molecular science could help to solve this issue.

## Figures and Tables

**Table 1:** Published PIF4-interacting proteins (until July 2021).

Gene Number	Annotation	Category	Reference
AT2G18790	PHYB	Red light photoreceptor	[6]
AT4G08920	CRY1	Blue light photoreceptor	[7]
AT5G53160	PYL8	ABA receptor	[8]
AT1G01360	PYL9	ABA receptor	[8]
AT1G75080	BZR1	Transcription factor in BR signaling	[9]
AT3G44450	BIC1	Regulator of CRY activity	[10]
AT1G30330	ARF6	Transcription factor in auxin signaling	[11]
AT2G01570	RGA	GA signaling repressor	[12]
AT2G46340	SPA1	PhyA signaling repressor which has Ser/Thr kinase activity	[13]
AT2G47890	BBX11	B-box family transcription factor	[14]
AT2G34640	HMR	Transcription activator	[15]
AT2G25930	ELF3	Transcriptional regulator repressing clock- and growth-associated transcription factors to regulate the circadian rhythm and hypocotyl elongation	[16]
AT5G61380	TOC1	Transcription factor	[17]
AT5G24470	PRR5	Transcription factor	[17]
AT5G02810	PRR7	Transcription factor	[17]
AT2G46790	PRR9	Transcription factor	[17]
AT3G50330	HEC2	Transcription factor	[18]
AT4G37580	HLS1	Transcriptional regulator	[19]
AT2G40360	BOP1	E3 ubiquitin ligase	[20]
AT2G41370	BOP2	E3 ubiquitin ligase	[20]
AT3G28910	MYB30	Transcription factor	[21]
AT1G43850	SEU	Transcriptional regulator	[22]
AT5G60970	TCP5	Transcription factor	[23]
AT3G02150	TCP13	Transcription factor	[23]
AT5G08070	TCP17	Transcription factor	[23]
